# Experiential-sensorial adaptive system model for hospitality based on 360° VR videos and case-based reasoning

**DOI:** 10.1371/journal.pone.0299387

**Published:** 2024-03-11

**Authors:** Luis Alfaro, Claudia Rivera, Jorge Luna-Urquizo, Francisco Ayala, Lucy Delgado, Elisa Castañeda

**Affiliations:** Universidad Nacional de San Agustín de Arequipa, UNSA, Arequipa, Perú; Sunway University, MALAYSIA

## Abstract

In this work, an adaptive software architecture is proposed for the generation of experiences for hotel promotion and marketing, based on Case-based Reasoning (CBR) that uses the attributes and user characteristics and immersive 360° videos. Considering that immersion in virtual reality (VR) environments can trigger responses in various dimensions, such as affective, cognitive, attitudinal, and behavioral dimensions, these dimensions are evaluated in immersive environments with 360° videos. To validate the results obtained with the software architecture, a quasi-experimental study was conducted through the evaluation of the experience, consisting in the visualization of the environments of a boutique hotel, with a sample of a randomly selected group of young people. The contribution of this work lies in the use of 360° VR videos, for the visualization of the hotel characteristics and environments according the user profiles, to evaluate the affective, cognitive and attitudinal and behavioral responses and their influence on the booking intention and attitude. Finally, conclusions and recommendations for future work have been established.

## Introduction

Peru has become an important tourist destination in South America and the world, as it has diverse recreational alternatives including visits to archaeological sites of the pre Inca and Inca cultures, as well as those of the Spanish viceroyalty, experiential tourism, gastronomic tourism, beach tourism, tourism in the Amazon jungle and Andean highlands, and tourism to hot springs centers, among others, activities that have become a composite business, providing diversified services that include accommodation, food, beverages, hot springs and entertainment. This condition demands that the actors in the national hotel business survive in a very fierce competition. In Peru, many companies have invested in the construction or renovation of hotels and various multi-service centers, increasing the interest in creating “Experiences” for customers and particularly for those users of the service sector and hotels.

Guests’ experiential outcomes during their hotel stay have been increasingly valued as important for planning and managing leisure services, as well as for understanding guest behavior and profiles. Current marketing strategies focus on ensuring and improving customer service, loyalty and purchase intent [1].

The Experiential Marketing approach associated with immersive VR technologies, including 360° technology, enables the design of applications for virtually visiting hotel facilities, services, and the surrounding landscapes. This is achieved using metaphors that, as described in reference [2], can be implemented through simulation, interaction, and reification techniques utilizing sensory stimuli and images. The primary focus is on enhancing experiences, making them closely resemble real-world experiences.

The techniques of Artificial Intelligence (AI) used for designing flexible and adaptive web systems focused on promoting service provision and product sales, such as hotel services, educational services, among others, when combined with sensory Experiential Marketing techniques and immersive VR technologies, provide users with direct experiences with low linguistic and computational mediation. These systems incorporate attributes of both explicit and tacit knowledge related to hotel services and the experiences they offer.

In this context, we propose, develop, and validate an architecture for a sensory-experiential marketing system based on 360° VR technology. VR employs virtual environments that allow users to move freely within the virtual space, while 360° VR offers spherical/panoramic experiences [3]. 360° immersive video has gained popularity in contemporary applications due to several studies indicating that it enhances perception.

This proposal, based on 360° VR technology, aims to create spherical/panoramic experiences to enhance the perception, credibility, and realism of environments. The use of real-world videos and photographs adds greater fidelity to the experiential environment [4]. Users can visualize their surroundings more clearly and in detail, resulting in a more enjoyable experience [5]. As Wu and Lin pointed out [6], it is possible to combine 360° videos with VR to achieve a better sense of perception and presence.

One functionality of the 360° VR model is to divide videos into segments that are then used for composing virtual tours based on the recommendations of the CBR component. This component takes into account user profile requirements, and the resulting virtual tours are viewed using 360° VR components. A second component based on CBR recommends the degree of immersion required for tour visualization, based on the user’s device resources and potential marketing strategies to be applied, a proposal that contributes to the originality of the model. For Wan et al. [7], when utilizing VR for promotional purposes, it is essential to include details of the target context. This is because various research studies have reported differences in the effectiveness and impacts of VR [8]. In this perspective, we considered dimensions of cognitive load, affective responses, attitudinal factors, and behavioral aspects to evaluate the visualizations [9] and their influence on booking intentions and attitudes toward the hotel.

The article consists of several sections. In Section II, the current state of the field is discussed. Section III focuses on the characteristics of the software model and the CBR engine. The methodology employed in the study is explained in Section IV. Section V provides a detailed description of the tests conducted and presents the results. Finally, in Section VI, the conclusions drawn from the study are presented along with recommendations for future research.

## Literature review

### Marketing and experiences

Experiential marketing is focused on creating enriching experiences for customers by communicating marketing actions that target the various senses of the consumer. The goal is to influence their preferences and subsequent decisions regarding a product, service, or brand. According to Alagöz and Ekici [10], “the primary component of experiential marketing is the experience, with the aim of encouraging consumers to actively engage and respond in the buying process, allowing various sensations and perceptions to emerge from their experiences.” There is currently a growing interest in understanding the experience that sensory marketing generates in consumers [11].

The approach of experiential marketing considers consumers as sensitive and rational human beings eager to explore objects, experiences, and sensations, rather than merely as consumers seeking to fulfill their needs through actions that yield utility. The expected outcome is that consumers respond to stimuli, exhibit active purchasing behavior, and enjoy entertaining sensations and experiences [10].

Numerous studies have been conducted on consumer behavior and sensations, with a focus on the senses of smell, hearing, touch, taste, and especially sight, making them emerging areas of research in marketing and sensory perceptions [12]. Marketing actions generate sensory experiences, and in various research studies, these experiences are linked to immersive experiences in VR environments, with a focus on their utilization of the various senses of the consumer. Sensory marketing is increasingly focused on creating memories through virtual experiences. Expectations are also formed based on prior experiences with the service, explicit and implicit communication, and word-of-mouth recommendations. Nowadays, the unconscious role of the human mind in decision-making is considered more relevant, suggesting that purchasing decisions have an emotional component.

Lastly, VR technologies can be integrated into various touchpoints between the company and its customers, aiming to support, enhance, or create new memorable experiences, ultimately offering value-added propositions throughout different stages of the customer’s purchasing process.

### Marketing strategies for hotels

Various studies aiming to improve hotel occupancy rates are diversified as follows:

#### Marketing strategies

Some traditional strategies of Peru’s hotels are focused on the following:

- Product strategy: focused on the rental of hotel rooms, bungalows and meeting rooms and other facilities. Environments provided by standard hotels, such as rooms equipped with beds, television and wifi, air conditioning, and some of them including a swimming pool.
- Pricing strategy: based on competition, there is a wide availability of offers, promotions and preferential attention to members of groups of guests, and even psychological prices.
- Advertising strategy: Advertising strategies use advertisements through the Internet, brochures, digital marketing, social networks and TV, among others.
- Location strategy: Hotels in Peru are strategically located in urban areas, rural areas, the Amazon jungle, in some cases close to shopping areas, craft fairs, historical monuments, beach resorts, casinos and leisure centers.
- Service strategy. Peru’s hotels in the 3-star and above segment provide adequate services, such as clean rooms, parking, comfortable lobbies, a varied breakfast including typical Peruvian cuisine, and friendly and responsive staff.
- Physical environment: The interior design includes modern buildings, as well as old buildings that have been refurbished and are well maintained. The environments around the hotels are diverse, including urban, archaeological, rural, Amazonian landscapes, beaches, etc.

These traditional strategies are the most commonly used by hotel business owners and entrepreneurs.

#### Experiential—Sensorial marketing strategies

Experiential marketing allows for activities that engage the senses and lead to action and experimentation. It involves designing an environment oriented toward interactions with stakeholders, especially collaborators, and aims to enhance the quality/price/promotion attributes of products or services [13]. It is also essential to identify its contributions towards marketing action and the service itself. Services are characterized by attributes that allow evaluation by various agents, including the business itself, customers, and suppliers. The experience is more holistic and can be internally evaluated by each customer [14]. Unlike the economic rationality of services, experiential marketing adds value through hedonistic and memorable sensations [15]. To achieve this, experiential marketing focuses on creating cognitive, sensory, relational, and emotional experiences for consumers [16].

Otto and Ritchie [14] defined the dimensions of the customer experience, developing a “Service Experience Scale” focused on tourism and leisure experiences. The four dimensions are hedonism, recognition, involvement, and tranquility. Pine and Gilmore [17] later proposed an adapted scale with four dimensions:

- Prior knowledge and education of the tourist.
- Aesthetics of the environment.
- Entertainment.
- Visitor’s need for escapism

Tourism experience marketing is focused on meeting the needs of tourists to live experiences based on tourism products categorized into sensory, emotional, action, and thought experiences [18]. Govindan [19] argues that the design of tourism product experiences should take into account the different perceptions of the various senses in the experience. Tian [20] maintains that the tourism experience has a phenomenological perspective, involving a significant process in which time and space are intertwined. A review of the literature reveals that many scholars have conducted research on experiential marketing in the tourism industry, with most of them analyzing experiential marketing strategies. Building on previous research, this article proposes strategies for applying them in this case.

For [1], hotel companies require a strategy that can increase customer satisfaction in order to raise the occupancy rate of the installed capacities, proposing the following for this purpose.

- Sensory experience: The sensory experience strategy could be carried out by promoting local culture and customs, such as welcoming guests with dances and music typical of the hotel area. Focusing on the sense of sight could include pictures of tourist destinations, displaying decorations with local handicrafts at strategic points in the hotel or rooms, and staff could also wear uniforms with costumes typical of the hotel area. The focus on the auditory sense can include music and songs typical of the hotel’s region. The approach to the sense of taste can include the diversity of culinary dishes of the rich Peruvian gastronomy and of the regions in the restaurant services. With the focus on the different senses, the aim is to impact guests with unforgettable impressions so that they book and return in the future.
- Feel the experience: Hotel identity can include the use of items such as sandals, soap, shampoo, toothpaste and toothbrushes to make it easier for consumers to remember the hotel’s identity. Service reputation can be enhanced by providing quality services, offering convenience in management procedures, friendly and pleasant services and maintaining safety in the hotel environment.
- Think of the experience: Give consumers surprises or a surprise gift, associated with birthdays, company anniversaries or other important dates and even commemorating some holidays.
- Act experience: Focus on aspects related to the hotel’s identity, the attractiveness of the interior and its decoration, the food menu, the music, the hospitality of its employees, which would be imprinted in the memory of consumers.
- Experience storytelling and storydoing: Combining the above experiences can bring value in consumer satisfaction, i.e., with five sensory experiences, behavioral experience, knowledge experience and physical experience.

Furthermore, marketing scholars established the concept of Customer Brand Engagement (CBE), considering cognitive, affective, and behavioral dimensions [21]. Affective engagement occurs when a positive relationship is established with customers, considering benefits and key features or providing them with opportunities to have memorable and enjoyable experiences to enhance their perceptions, preferences, attitudes, and behaviors toward the brand. Behavioral engagement emerges when active participation is achieved, facilitating and improving the desire to purchase or use a brand’s product or service. Finally, cognitive engagement triggers a conscious state of attention that can impact brand awareness, retention, and absorption.

If CBE is situated in an enriched environment, it enables immersive interactivity, requiring the dimension of social interaction to capture the social engagement of the brand. Social engagement involves sharing the brand, experiences, knowledge, and support. Thus, in a context of 360° VR experiences that incorporate the social dimension, CBE includes a motivational mental state that results from the affective, behavioral, cognitive, and social responses of the consumer, which in this study will be mediated by 360° VR.

### VR in marketing and advertising

Burdea and Coiffet [22] define VR as “the use of a 3D computer-generated environment in which the user interacts and navigates in a real-time simulation perceived by the ‘five senses’ of one or more users.” For Gutiérrez et al. [23], the elements of VR are: simulation, sensory immersion, and implicit interaction. Additionally, VR is associated with a three-dimensional world, in which users interact with the virtuality of an environment, allowing for the construction of knowledge with low symbolic linguistic and computational mediation. Experiences of this kind may not be available in the real world, thus having enormous potential in marketing and advertising, education and training, considering concepts such as “Dimension,” “Transduction,” and “Reification” [24]. VR can also manifest as avatars, which can interact with each other and with other elements of the virtual world.

According to [25], new technologies offer enormous potential to tourists, contributing to the search for their destinations and the optimization of the experiential process, both in space and time, and improving the co-creation of value with stakeholders in the sector. Mobile technologies provide the possibility for tourists to participate simultaneously in real and virtual experiences [26]. In [27], it is argued that technological solutions for VR-based applications can be fully immersive, semi-immersive, or non-immersive, affirming that the more immersive the solution, the more complex the implementation of content and technology will be, due to the devices used. Head-mounted displays (HMDs) used by users for VR-based tourism marketing activities are considered a good solution due to their immersion capability and cost-effectiveness.

The integration of technologies provides customers with value-added propositions that create optimal experiences through the combination of physical and virtual touchpoints [28]. The use of VR technologies allows consumers to play more dynamic and autonomous roles in their experiences [29], contributing to experiences and perceptions with added value. Research in VR generally focuses on evaluating customer immersion and presence perceptions [30], as well as narrative transportation or story plot establishment, which could be complementary to these elements [31]. Narrative transportation is based on the cognitive processes experienced by the customer as a result of emotional immersion in a story with characters and plot [31]. Many studies establish that content with narrative structures could enhance the sense of presence, due to the effects of sensory signal stimuli. Presence as an experience in a virtual world is as if it were authentically happening in the real world, allowing for the evaluation of consumer visualization, taking into consideration the effect of the environment [21]. Experiential immersion provides responses based on visual and aesthetic elements [32]. The sense of reality created by these feelings in VR environments is crucial because consumers cannot physically touch objects (due to lack of haptic feedback resources).

Information obtained through AR/VR/MR technologies stimulates consumers’ imagination before, during, and after purchase [33]. Omnichannel experiences are enabled by these technologies, which are enhanced using different online and offline touchpoints for consumers [34]. VR and 360° VR technologies are the tools used in the Adaptive Experiential and Sensory Marketing model proposed in this work, enabling interactions considering users’ profiles and interests to generate personalized virtual experiences and tours with experiences and information from a hotel located in a specific environment.

### 360° VR in hotel tourism marketing

360° video is defined as a technology that “allows viewing real-life content synthetically or in 360°, creating experiences that stimulate visual and other senses, experiences used in planning, information exchange, management, marketing, education, entertainment, preservation, or accessibility of cultural heritage, whether before, during, and/or after the journey” [27]. It is used to generate real-time immersive experiences very close to reality, using 3D and 360° VR technological resources [35]. It provides good control over visualizations and experiences, which are interactive and of higher quality and intensity than standard videos, thanks to the incorporation of immersive attributes [36].

According to [37], 360° VR can represent potential tourist destinations more convincingly than traditional videos due to the close resemblance of the virtual world to the real one. These concepts are applicable to both hedonistic and utilitarian users who prefer first-person VR-based applications. It is important to understand both extrinsic and intrinsic motivations and their impact on user satisfaction in VR environments and their influence on travel intention decisions, which is the focus of this research.

To validate the results obtained from the proposed architecture, the research questions are:

- What is the methodology to be used for the development of the experiential-sensory software model for hospitality based on 360° VR videos and CBR?
- How will the intelligent component of CBR be validated?
- How do the visualizations of traditional photographs and videos compare to those of 360° videos in VR aplicaciones, in relation to: (1) affective responses, (2) attitude/behavior responses, and (3) cognitive load, pertaining to efforts required by tasks and experiences using technology?

### Case-based reasoning (CBR)

CBR is an AI approach used for problem-solving, identification, learning, reasoning, decision support, and fault treatment. It is a problem-solving approach based on experience that reuses knowledge by considering experiences and solutions from similar cases to solve new problems.

It includes a previously stored case base. According to Bergmann [38], similar cases may have similar solutions obtained from many cases, adapting a particular solution to address the new problem, and the new solution can be stored in the Case-Base to enhance its competence [38]. CBR operates in a cycle that includes four stages: retrieve, reuse, revise, and retain.

## Software architecture proposal

Architecture is focused on generating experiences based on experiential and sensory marketing strategies and providing different stimuli to potential users, expecting that customers respond to stimuli that induce active buying behavior after enjoyable and pleasurable experiential encounters [10]. Verhoef et. al [39] argue that the customer experience is based on a multidimensional construction process of holistic characteristics, involving social, physical, cognitive, affective, and emotional responses.

**Fig 1:**
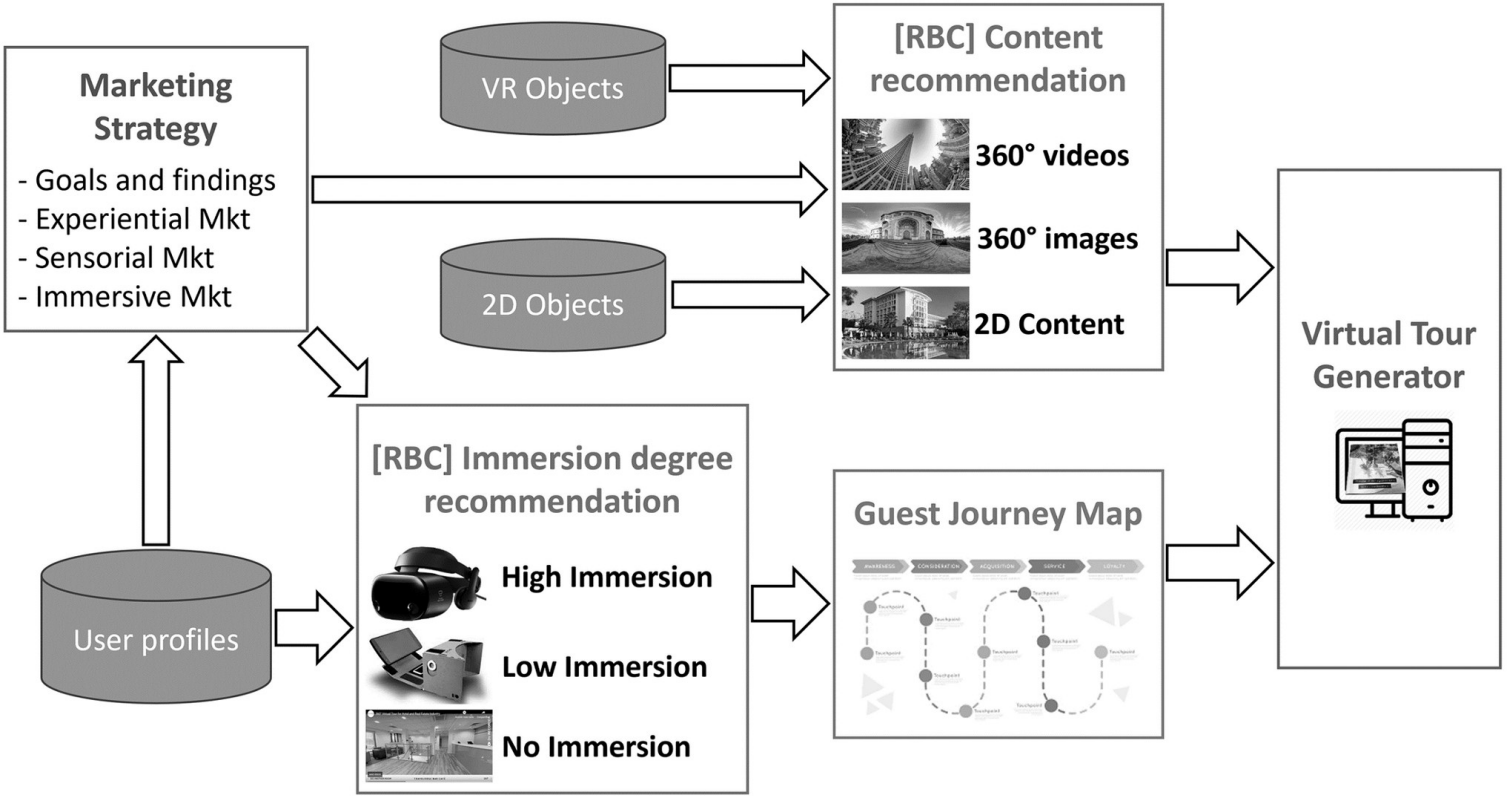
Software architecture.

The architecture is composed of 5 modules, as described in Fig 1.

For the implementation and development of virtual content generation, the 4+1 architectural view model was used, which describes software system architectures using multiple simultaneous views. The model consists of 5 components, including an intelligent component that adapts to customer profiles, which is described in the following section.

### Immersive marketing strategy

In this module, the strategy that is required for the generation of virtual content is generated, and will be implemented, regarding the determination of types of experiences, virtual content, and objects, as well as the creation of static images, videos with VR resources, with 360° technology, texts, gifs, etc. It is in this module that strategies oriented to the senses of sight, taste and hearing should be incorporated according to the discussion in the experiential marketing strategies section.

### Virtual content generation

The module incorporates tools and provides the structure that enables the generation of virtual objects, including 360° videos, which are segmented and then composed into virtual tours according to the profiles provided by the CBR. It also includes information for access through the required hot points for dynamic interactions available for experiential-sensory marketing strategies. These strategies encompass videos and images of dances, typical costumes, culinary art, musical instruments, and other elements.

Additionally, it allows for the simulation, evaluation, testing of virtual content, and the storage of resources related to the solution’s behavior concerning different profiles and adaptation parameters used by the CBR. These resources are required by customers in the process of seeking information and exploring hotel offers, infrastructure, facilities, cultural and leisure activities, as well as the hotel environment.

### CBR content recommendation

**Fig 2:**
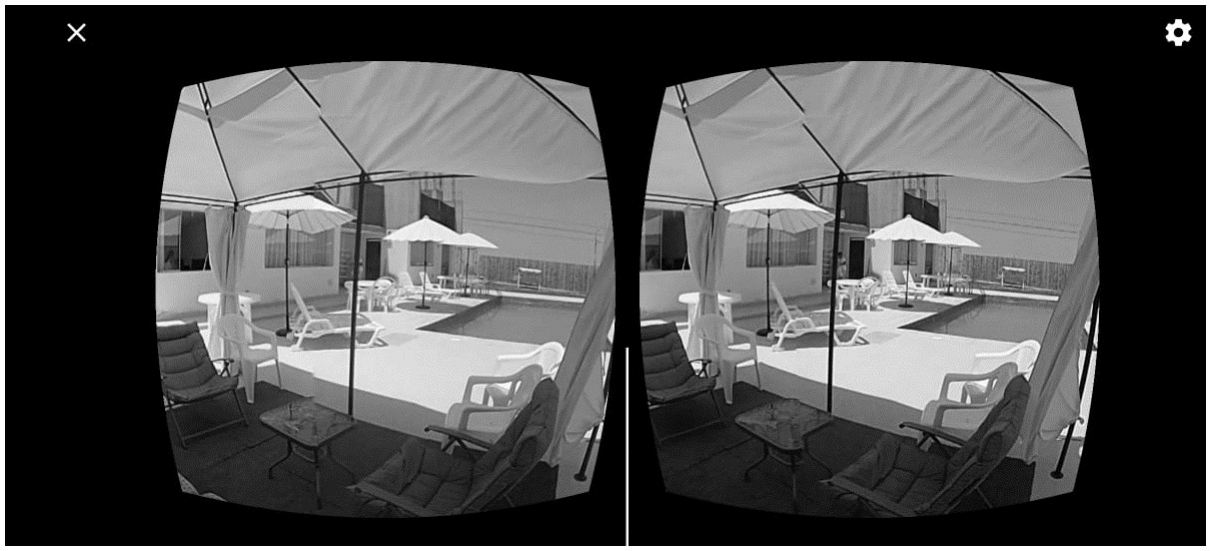
Virtual tour.

It is focused on the logic of the adaptive system and executes the established marketing strategy. It also performs user profile identification, enabling adaptive virtual presentation and navigation, allowing the visualization of facilities, services, and environments. It includes a component based on CBR that should establish tours based on user profiles and the availability of immersive devices. The objects within the Hotel Marketing system will be dynamically presented to clients or system users according to their demands through the interface shown in the Fig 2.

The proposal employs 360° VR videos, allowing users to embark on virtual tours of the hotel’s facilities, surroundings, and attractions. These tours are the outcome of a composition that utilizes video segments obtained by the system to suggest tours based on recommendations from the CBR component. Users can enhance these tours by adding elements such as videos, images, soundtracks, etc., to create marketing strategies. These strategies enable users to explore using hot points. Each ‘case’ represents the outcome of a specific query conducted using a descriptor characterized by a set of attributes retrieved from the databases utilized by hotel companies, as detailed below:

- Id: identifier used for user route recommendations.
- Zone: location of the hotel, an important characteristic that can be in the city, the countryside or the beach.
- Motive: motivation of the user’s trip, which can be for work, holidays, etc.
- Services: services offered by the hotel that are required to be visualized through 360° images or videos and that may be considered of particular interest to the user at the time of the tour.
- Company: if the user is travelling alone or accompanied by a partner, family, friends, etc.
- Priority: a descriptor of a tribute or significant feature considered important by the user when choosing a hotel.

Six parameters were defined to establish the functionalities of the search engine, similar to the search cases of the CBR mechanism:

- Attribute name: name of attributes considered for the search.
- Search value: attribute values of the most approximate search case.
- Weightings: these are attributes assigned during the search, which were previously obtained based on the average opinions of hotel management and marketing experts, as well as potential users, as shown in Table 1.
- Terms: this represents the search form for each attribute, and is defined for searching for equal values, greater than, less than, closure, etc.
- Scales: these are the mathematical representations, which can be logarithmic, linear, etc., of how to search for the differences between the stored cases and the new case.
- Search options: this is a method for returning search results, where it was predetermined to return the closest values.

**Table 1:** Attribute table.

Attribute	Weight
Zone	15%
Motive	24%
Services	17%
Company	20%
Priority	24%
**Total**	**100%**

The search results are ordered or ranked in a way that prioritizes cases closest to the baseline. These cases are displayed in the immersive interface as recommendations that take into account the preferences entered previously. If, after the search, the user decides to follow a recommended route, a new case will be created in the Case-Base based on the data entered by the user. This increases the database of cases, allowing for improved subsequent searches by returning cases that are closer to the user’s preferences.

### RBC immersion degree recommendation

The objective of the module is to determine the recommended degree of immersion for tour visualization, based on the user’s device resources and the possible marketing strategies to be applied. To achieve this goal, a CBR mechanism is proposed, which considers the various attributes corresponding to the user’s profile and selects the most appropriate resources so that the marketing strategies are as effective as possible.

To do this, the following attributes of the user’s profile are considered:

- Age: various studies focus on the influence of age on the effectiveness of using immersive resources, making it an important factor to consider because its impact can be relative and will also depend on various factors such as the health status of a particular individual.
- Experience with immersive devices: this attribute allows for adjusting the recommended degree of immersion based on the user’s previous experiences, aiming to facilitate their use and adoption.
- Time: maximum time the user is willing to invest in the activity.
- Available devices: the type of available devices will determine the format or type of resources that can be used in experiences, serving as a highly restrictive factor.
- Languages: refers to the languages the user is proficient in, prioritizing resources whose content is available in these languages for better utilization of available resources.
- Health factors: high levels of immersion are generally not recommended for individuals with anxiety disorders, nausea, visual fatigue issues, among others. These health factors serve as restrictions on the type of recommended resources, although their impact may vary depending on age and other factors.

For marketing activities or resources, they are defined in terms of the following attributes:

- Resource Type: related to the degree of immersion utilized by the resource, they can be non-immersive, semi-immersive, or immersive, allowing for the exclusion of some resources depending on the user’s available devices and their experience in using immersive devices.
- Duration: the estimated number of minutes required to complete the activity. It should be checked whether the user is accustomed to completing long-duration activities (more content-heavy) or prefers completing specific tasks.
- Language: the language in which the content and/or interface of the resource are available, or indicating if the resource is available in more than one language, so that it can be configured and selected according to the user’s preferences.

For the search for similar cases implemented in the CBR, it is executed based on the following six (6) parameters:

- Attribute Names: identifiers of the attributes to be compared by the search function.
- Search Values: these are the values considered in the search case, which will serve as the basis for comparisons.
- Weights: this parameter represents the importance of each attribute when conducting the search, so the CBR will prioritize those attributes with higher weights. These weights were obtained based on the average opinions of university professors, researchers, and some potential users, as shown in Table 2.
- Method: defines the method of comparison for each attribute and can restrict the search to identical values, greater or lesser values, nearby values, etc., depending on the type of attribute and the search values used.
- Scales: this parameter represents how the difference between cases and the search case will be searched mathematically. It can be searched linearly, logarithmically, etc.
- Search Options: this parameter represents how the search results will be ordered. The default option is to return the closest values, but it can be configured to return the results in reverse order.

**Table 2:** Relative weights assigned to the attributes of the cases in the RBC.

Attribute	Relative weight
Age	6%
Level of knowledge	20%
Experience	20%
Time	12%
Available devices	24%
Language	12%
Health factors	6%
**Total**	**100%**

To calculate the similarity function, the Euclidean distance is used, where a lower value represents a shorter distance and therefore greater similarity between the analyzed values.

The search results are displayed in an array of cases ordered, showing the case that most closely resembles the search case first, as recommendations for the user, who will interact with the system in a textual or voice manner. If the user selects one of the recommended cases, a new case will be created with the course information and the user information. This will help improve the results of subsequent searches and enable recommendations that are closer to the needs and preferences of the users.

For the implementation of the RBC, the API of the FreeCBR tool (open source) was used as a base. It’s worth mentioning that this model combines various AI techniques.

### Guest journey map

The module is focused on the available devices for implementing the marketing strategy, whose features determine the degree of immersion for the user. The implementation of the proposal in real cases involves using techniques like Journey Maps [40], defined as a human-centered qualitative design to focus and visualize the actions, feelings, thoughts, or the experience of a customer or a guest. A Journey Map guides the coding quantitatively by dividing an agent’s “journey” into steps [41] and storytelling concepts [42], utilizing short audiovisual recordings to capture the viewer’s attention and convey information clearly and swiftly, influencing their decision-making considering emotions and feelings, focused on the experiential and sensory approach in this work. On the other hand, the Storydoing technique [43] involves telling a story consistent with the behavior of the user so that it goes beyond a mere narrative. In this context, storydoing enhances consumer immersion through various digital techniques within the omnichannel framework. Immersive marketing can use storydoing as a source of development, as emphasized in this work. This original proposal of an adaptive intelligent model is focused on various aspects of experiential marketing, AI techniques, and methodologies and technologies of Immersive 360° VR, which are part of this software architecture. It aims to contribute to the promotion of hotel services, facilities, and environments.

## Methods

The development and validation methodology to be followed is outlined as follows:

### Development of the model

The development of the model comprises seven stages:

1. Establishment of requirements and gathering of information related to user experiences with hotel executives.
2. Mapping the guest journey and analyzing the experience with experts in management and marketing.
3. Capturing 360° videos and images of the boutique hotel, environments, and surroundings, as well as searching and selecting videos, images, and musical tracks to create experiential-sensory evidence related to the culture and tourist and gastronomic attractions of the area.
4. Creating and designing user interface prototypes for the immersive application.
5. Implementing the immersive visualization layer using Unity, specifically tailored for VR immersive headsets.
6. Implementing the CBR engine and a REST API.
7. Conducting functionality tests, validation, and effectiveness tests of the model using the TLX-NASA method, following the work of Söderlund [44] and Slevitch and Oh [45].

### CBR engine

The CBR Engine operation begins with the generation of a new journey based on the search for a recommendation, using input from a new case that contains the following data: destination area, purpose of the trip, required services, companions, and search priority.

The CBR configuration defines the terms, scales, and weights for recommendation searches. At the end of the evaluation, a sorted list based on similarity, determined by the accuracy value, will be returned in all cases. Identical cases will be removed to avoid duplicate results, and the information obtained will be used by the CBR. The web server uses the list of journey IDs to retrieve the information that will be displayed to the user.

In the event that the user decides to follow one of the tours recommended by the model, it is considered that the case aligns with their interests. The parameters for the search and the selected journey are stored as a new case in the Case-Base. This allows for their use in subsequent searches, enhancing the precision of future recommendations.

### Methodology for validating the effectiveness of the model

The research conducted in this study is exploratory and descriptive in nature, following a non-experimental and cross-sectional design. The adopted approach is quantitative, utilizing adapted questionnaires from [30] to obtain affective responses, [44, 45] to gather attitudinal responses, and [46] to evaluate cognitive load. The NASA Task Load Index (TLX) test was used for assessing cognitive load. The sampling method employed was random, with 203 questionnaires distributed among a young population. The statistical SPSS package was used for data processing. The study focused on a website of a boutique hotel located in Arequipa, Peru, which included images of rooms, facilities, services, and the surrounding environment. For the 360° VR video test case, the VR HMD (Virtual Reality Head-Mounted Display) device was used, which allows for immersive experiences solely based on a smartphone [47]. Participants in the VR group were provided assistance in wearing the devices and gave verbal consent for the use of their images in the study.

Authorization and approval of the protocol for conducting the experiment were requested from the ethics committee of the Universidad Nacional de San Agustín de Arequipa, within the framework of the research project: “Experiential-Sensorial adaptive system model for Hospitality based on 360° videos and CBR”. The response received was as follows: “After reviewing the mentioned project, this Committee concluded the following: The study does not violate any Ethical Principle, so Approval from this Committee is not considered necessary”.

The experiment was carried out in a variety of locations and environments. Subjects participated individually with an average duration of 5 minutes, being asked to imagine themselves in a situation of selecting a hotel, considering the location and motivations included in the study. Verbal authorization was requested for the use of their images for the purpose of documenting the study. A Google questionnaire was completed after the experiment to answer questions evaluating behavioral, cognitive, affective, and attitudinal responses, including demographic information. The test was conducted over a period of 1 month.

Several researchers argue that literature on consumer behavior allows for establishing that affective, cognitive, behavioral, and attitudinal responses can contribute to understanding behavior prediction, user consumption habits, and decision-making.

To validate the results obtained with the architecture, the following questions are posed:

How to compare videos of web page visualizations with content of photographs and videos with adaptive 360° VR tours in terms of: (1) Cognitive load, which involves efforts related to technology usage and tasks derived from user experience? (2) Behavioral/attitudinal responses and (3) Affective responses?

VR can trigger experiences in dimensions such as cognitive, sensory, attitudinal, affective, and behavioral [9].

#### 1. Cognitive response—CLT [48]

The model proposes that there is a limited attention capacity [49], serving as a framework for understanding 360° VR visualizations. Images of hotels visualized with 360° VR may involve greater cognitive/perceptual load than traditional image visualizations, content, and objects on web pages, allowing the following hypothesis to be established:

**Hypothesis 1a**: Hotel visualizations in 360° VR environments do not generate a higher cognitive load compared to traditional image, content, and object visualizations of similar scenes contained on websites.
**Hypothesis 1b**: Hotel visualizations in 360° VR environments generate a higher cognitive load compared to traditional image, content, and object visualizations of similar scenes on websites.

#### 2. Affective response

Various researchers report that experiences in VR environments can trigger emotional conditions, such as increased heart rate, elevated blood pressure, skin conductance responses, and changes in respiratory rate [50]. Because VR activates the senses, affective responses may be stronger compared to traditional images. Immersive VR influences the presence of sensory stimuli, fostering intense emotional experiences. It has also been reported that VR evokes affective responses, including pleasure, excitement, and feelings of frustration [51]. Therefore, it is assumed that hotel videos with 360° VR may provoke stronger affective responses compared to traditional photos and videos contained on hotel websites, leading to the following hypotheses:

**Hypothesis 2a**: Hotel environments visualized with 360° VR do not generate stronger affective responses compared to traditional visualizations of images, similar content, and other scenes on websites.
**Hypothesis 2b**: Hotel environments visualized with 360° VR will generate a stronger affective response compared to traditional visualizations of images, similar content, and other scenes on websites.

#### 3. Attitudinal and behavioral responses

360° VR videos can influence customer attitudes and behavior. Van Kerrebroeck et al. [52] reported that consumers have more positive attitudes, loyalty, and satisfaction after participating in VR experiences. Studies by Stoyanova et al. [53] suggest that presence and representational richness (an important attribute for VR) can influence more positive attitudes towards a particular brand and higher purchase intention.

Considering this assumption, the established hypotheses are:

**Hypothesis 3a**: Hotel environment visualizations in 360° VR do not generate more positive attitudes compared to traditional visualizations of content, images of similar scenes on websites.
**Hypothesis 3b**: Hotel environment visualizations in 360° VR generate more positive attitudes compared to traditional visualizations of content, images of similar scenes on websites.
**Hypothesis 4a**: Hotel environment visualizations in 360° VR will not generate more behavioral attitudes compared to traditional visualizations of content, images, and other similar scenes on websites.
**Hypothesis 4b**: Hotel environment visualizations in 360° VR will not generate more behavioral attitudes compared to traditional visualizations of content, images, and other similar scenes on websites.

Affective responses to the visualizations were evaluated based on the Pleasure, Arousal, and Dominance (PAD) scale [30], which has been validated by various studies assessing affective responses. The scale is applied to physical environments and colors [31]. When applying the scale, it begins with the statement: “After viewing the hotel images, I feel…” followed by 18 bipolar items.

1. Adjectives focused on pleasure, in pairs: happy-unhappy, satisfied-dissatisfied, content-bothered, joyful-depressed, hopeful-desperate, relaxed-bored.
2. Adjectives focused on excitement, in pairs: stimulated-relaxed, excited-calm, frantic-calm, awake-drowsy, excited-unexcited.
3. Adjectives focused on dominance, in pairs: controller-controlled, influential-influenced, important-insignificant, dominant-submissive, unrestricted-restricted.

For the evaluation of the bipolar pairs, a 7-item semantic differential scale is used, ranging from 1 to 7 [31]. Behavioral intentions focused on the hotel domain were assessed with a 7-item scale adapted from Slevitch and Oh [45], which includes items focused on attitudinal aspects such as:

1. Is there a possibility that you would decide to book the hotel you saw?
2. How satisfied would you feel deciding to stay at the hotel?
3. Does the visualized hotel match the price range of your expectations?

The questions focused on behavior are as follows:

1. What is the likelihood of booking a hotel of this type?
2. What is the likelihood of sharing positive things about the hotel with others?

The assessment of cognitive workload can be carried out using NASA’s TLX tests, focusing on subjective mental workload [54], considering the cognitive demand of the task [46]. NASA TLX evaluates workload in six areas: physical demands, mental demands, temporal demands, effort, as well as performance and frustration, which are assessed using the differential scaling method. The validity and reliability of the method have been confirmed in several studies.

The consistency of the survey questions for both groups was validated using Cronbach’s alpha method, yielding satisfactory results as shown in Table 3.

**Table 3:** Reliability statistics.

Cronbach’s alpha	Cronbach’s alpha based on standardized elements	Number of elements
.830	.832	10

## Data analysis and discussion of results

### Analysis of the validating the effectiveness of CBR engine

The test was conducted using the Case Library Subset Technique (CLST) for CBR validation [55], focusing on selecting a subset of the Case-Base for use in evaluating the retrieval rate effectiveness. The parameters used by the technique are as follows:

- Results Acceptance Criterion (RAC): maximum relative error allowed, obtained by comparing with the gold standard. For this research, an RAC of 15% was defined.
- System Validation Criteria (SVC): threshold to determine whether, after evaluating the subset of cases in the system, the current case is considered valid. In CBR, accepted cases must have a threshold of 75%.

The evaluated cases can yield two types of results: “SUCCESS” for those that pass the test, or “FAILURE” for those that do not pass.

The acceptance rate obtained was 100% retrieval and 88% adaptation success in tests conducted with 50 cases, as shown in Table 4. This indicates that the proposed CBR model is valid, considering the previously established SVC threshold of the CLST technique.

**Table 4:** Results of retrieval and case adaptation tests.

N°	Recovery	Result	Adapt	Result	N°	Recovery	Result	Adapt	Result
1	100.00	Success	100.00	Success	26	100.00	Success	86.26	Success
2	100.00	Success	100.00	Success	27	100.00	Success	86.26	Success
3	100.00	Success	100.00	Success	28	100.00	Success	86.26	Success
4	100.00	Success	100.00	Success	29	100.00	Success	86.26	Success
5	100.00	Success	100.00	Success	30	100.00	Success	86.26	Success
6	100.00	Success	61.27	Failed	31	100.00	Success	100.00	Success
7	100.00	Success	100.00	Success	32	100.00	Success	100.00	Success
8	100.00	Success	61.27	Failed	33	100.00	Success	100.00	Success
9	100.00	Success	100.00	Success	34	100.00	Success	100.00	Success
10	100.00	Success	61.27	Failed	35	100.00	Success	100.00	Success
11	100.00	Success	100.00	Success	36	100.00	Success	100.00	Success
12	100.00	Success	100.00	Success	37	100.00	Success	56.13	Failed
13	100.00	Success	100.00	Success	38	100.00	Success	100.00	Success
14	100.00	Success	100.00	Success	39	100.00	Success	51.01	Failed
15	100.00	Success	100.00	Success	40	100.00	Success	100.00	Success
16	100.00	Success	100.00	Success	41	100.00	Success	100.00	Success
17	100.00	Success	100.00	Success	42	100.00	Success	56.13	Success
18	100.00	Success	100.00	Success	43	100.00	Success	100.00	Success
19	100.00	Success	100.00	Success	44	100.00	Success	100.00	Success
20	100.00	Success	100.00	Success	45	100.00	Success	100.00	Success
21	100.00	Success	100.00	Success	46	100.00	Success	100.00	Success
22	100.00	Success	100.00	Success	47	100.00	Success	100.00	Success
23	100.00	Success	100.00	Success	48	100.00	Success	100.00	Success
24	100.00	Success	100.00	Success	49	100.00	Success	100.00	Success
25	100.00	Success	100.00	Success	50	100.00	Success	100.00	Success

### Analysis of the validating the effectiveness of the model

For the sample, 312 clients were considered from a young population composed of 1,620 users, including professionals, graduate, and undergraduate university students. Each of the 2 tests included 156 participants per group. The results analysis is as follows:

For extraction, the Principal Component Analysis method was used, considering only 3 components. Varimax with Kaiser normalization was applied as the rotation method, and a correlation cutoff value of 0.5 was used. Based on the Bartlett’s test of sphericity (*p* < 0.0000), it was established that factor analysis was appropriate. Additionally, the Kaiser-Meyer-Olkin (KMO) test yielded a statistic of 0.838.

#### Cognitive load answers

The responses showed some differences between Test 1 and Test 2 in most items of the cognitive load dimension (refer to the 2D cognitive dimensions table available in [56]). Test 1 obtained significantly lower scores in the mental demand scale compared to Test 2, equivalent to 12.5%. The physical demand in the 360° VR visualization was 16.6% lower compared to the 2D visualization.

Temporary demand during the visualization had a difference of 13.5% between 360° VR and 2D. There was less effort in 360° VR visualization, equivalent to 8.3%, compared to the 2D version. The level of frustration was slightly lower in 360° VR, with a 4.2% difference compared to 2D.

#### Affective answers

Differences were recorded in the responses between Test 1 and Test 2 in most items related to the affective load dimension (refer to the 2D Affective Dimension table available online [56]). The highest statistically significant difference was 17%, corresponding to the female gender. There were differences in the responses related to how insecure, discouraged, irritated, stressed, and annoyed the participants felt in relation to the task. In the question about disgust and satisfaction, the results were lower by 4.9% in Test 1 compared to Test 2.

The 360° VR images received lower scores compared to the scores produced in 2D visualizations, with a -2.1% difference for the Animated-Abashed Scale. Participants who viewed in 2D obtained higher scores compared to 360° VR, with a 9.4% difference on the time-relaxed scale.

#### Attitudinal and behavioral responses

Differences in attitudinal and behavioral responses were observed between the data from Test 2 and Test 1. Participants in Test 2D expressed feeling more positive about staying in the hotel compared to those who viewed the 360° VR images, with a 29% difference. Participants who viewed 2D photographs showed a higher likelihood of booking a room in the hotel than those who viewed through 360° VR, with a difference of 25.9%. It is inferred that booking intentions would have a greater preference for 2D because the hotel’s website allows access to information about all the hotel’s services and surroundings, while 360° VR visualizations could be accessed through hotspots or by providing the user with tours based on their profile, showing only partial information about the hotel, facilities, and surroundings according to their interests. Therefore, further studies are needed to corroborate the obtained results.

The attitude and behavior dimension post-test established positive booking intentions and attitudes. Low levels in some responses in 360° VR may be linked to the video quality, which may lack visually appealing and representative features of the hotel facilities, or to the possibility that the selected hotel may not be interesting for the target audience of the sample, due to age, lifestyle, occupation, economic status, and inexperience as hotel users. Another aspect to consider could be the system response times during the experience, resulting from the composition of the tour, which includes elements for the implementation of Experiential-Sensorial Marketing strategies.

## Conclusion

A model of adaptive experiential-sensory marketing software architecture for 360° VR was proposed and developed to support hotel marketing strategies through segmentation and composition of 360° VR videos. To validate the effectiveness of the experiences with the model, adaptive 360° VR visualizations were evaluated and compared with 2D visualizations on the websites. Immersion and 2D image visualization can trigger experiences in the cognitive, affective, and attitudinal dimensions, which were assessed in this study.

The results of the study suggest that 360° VR views show differences compared to traditional 2D images on websites, but only in some dimensions. In the cognitive dimension test, it presented low cognitive load, facilitating information processing. In the affective dimension test, it elicited stronger affective responses, and in the attitude and behavior dimension test, positive attitudes and booking intentions were evident.

Furthermore, the results suggest that 360° VR users, visualizations, and the complexity of visual details produce various impacts on cognitive load, performance, and skill performance during the visit. Some low response levels may be due to video quality, which may not have included the visual appeal representative of the hotel’s facilities. It is also possible that the selected hotel may not be of interest to the target audience due to age, occupation, lifestyle, economic status, and lack of experience as hotel users. Another aspect to consider could be system response times when conducting the experience, which may result from the composition of the tour.

## Limitations and further research

The data were obtained in the city of Arequipa, Peru, considering potential hotel guests and students from UNSA-Arequipa and the Technological University of Peru (UTP). These participants might not accurately replicate real-world conditions.

The study had a limited scope of dimensions, excluding some that could be relevant.

In future studies, psychophysiological measures should be included to address possible shortcomings in using self-reported measures, which may not be the best way to gather data on affectivity.

The quality of the videos and the composition of sequences, as well as response times for navigation in 360° VR, could have affected the responses and should be considered.

Finally, for future research, the use of VR hardware for different levels of immersion should be considered, along with strategies focused on the sense of hearing.

## Supporting information

S1 File (XLSX)
